# Recombinant Adeno-Associated Virus: Efficient Transduction of the Rat VMH and Clearance from Blood

**DOI:** 10.1371/journal.pone.0097639

**Published:** 2014-05-23

**Authors:** Margriet A. van Gestel, Arjen J. Boender, Veronne A. J. de Vrind, Keith M. Garner, Mieneke C. M. Luijendijk, Roger A. H. Adan

**Affiliations:** Brain Center Rudolf Magnus, Department of Translational Neuroscience, University Medical Center Utrecht, University of Utrecht, Utrecht, The Netherlands.; Justus-Liebig-University Giessen, Germany

## Abstract

To promote the efficient and safe application of adeno-associated virus (AAV) vectors as a gene transfer tool in the central nervous system (CNS), transduction efficiency and clearance were studied for serotypes commonly used to transfect distinct areas of the brain. As AAV2 was shown to transduce only small volumes in several brain regions, this study compares the transduction efficiency of three AAV pseudotyped vectors, namely AAV2/1, AAV2/5 and AAV2/8, in the ventromedial nucleus of the hypothalamus (VMH). No difference was found between AAV2/1 and AAV2/5 in transduction efficiency. Both AAV2/1 and AAV2/5 achieved a higher transduction rate than AAV2/8. One hour after virus administration to the brain, no viral particles could be traced in blood, indicating that no or negligible numbers of virions crossed the blood-brain barrier. In order to investigate survival of AAV in blood, clearance was determined following systemic AAV administration. The half-life of AAV2/1, AAV2/2, AAV2/5 and AAV2/8 was calculated by determining virus clearance rates from blood after systemic injection. The half-life of AAV2/2 was 4.2 minutes, which was significantly lower than the half-lives of AAV2/1, AAV2/5 and AAV2/8. With a half-life of more than 11 hours, AAV2/8 particles remained detectable in blood significantly longer than AAV2/5. We conclude that application of AAV in the CNS is relatively safe as no AAV particles are detectable in blood after injection into the brain. With a half-life of 1.67 hours of AAV2/5, a systemic injection with 1×10^9^ genomic copies of AAV would be fully cleared from blood after 2 days.

## Introduction

Viral vector gene delivery is currently among the most widely used gene transfer tools for gene delivery in the central nervous system (CNS). Recombinant adeno-associated virus (rAAV) vectors mediate stable and long-term gene expression in both dividing and non-dividing cells without eliciting a significant immune response, making them an attractive viral vector system [1], [2]. rAAV vectors are easily designed because of the simplicity of the AAV genome. The inverted terminal repeats (ITRs) flanking the two viral genes *rep* (replication) and *cap* (capsid) of the wild type virus are the only elements necessary for virus replication and encapsidation. To design a rAAV vector, *rep* and *cap* are replaced by a promoter followed by the gene of interest or short hairpin RNA (shRNA) and subsequently provided *in trans* from a plasmid without ITRs.

For the determination of gene function in distinct areas of the brain it is of importance to optimize the AAV-mediated transfer and expression of genes or shRNAs. For this purpose AAV serotypes have been studied for their transduction efficiency in the brain. AAV2 is the most widely used serotype to transduce the CNS [1], [2]. Since different serotypes infect different and overlapping types of cells, the AAV2 vector has been pseudotyped in capsids from different AAV serotypes. AAV2/1, AAV2/5 and AAV2/8 have been shown to effectively transduce rat hypothalamus [3], striatum [4], [5], hippocampus [4], [6], [7], substantia nigra [4], [6], [8] and red nucleus [9].

This study focused on transduction of the ventromedial nucleus of the hypothalamus (VMH) in the rat brain, which is involved in energy homeostasis [10], fear [11] and female reproductive behavior [12]. AAV2/1, AAV2/5 and AAV2/8 transduction efficiency was compared in the rat VMH. We did not consider AAV2/2, because previously it was demonstrated that AAV2/1, AAV2/5 and AAV2/8 more effectively transduce neurons in rat hypothalamus, striatum, hippocampus and substantia nigra than AAV2/2 [3], [4], [8].

The use of different serotypes with their own receptor tropism may have implications for the viral particle biodistribution. An evaluation of the biodistribution for every serotype and for every route of administration is relevant to biosafety with respect to the use of AAV. Several AAVs have been shown to effectively cross the mouse blood-brain barrier after intravascular delivery [13], [14]. It is not known whether AAV delivery in the brain results in the introduction of viral particles in the bloodstream. This study therefore focused on AAV vector transfer to the blood after AAV2/1, AAV2/2, AAV2/5 or AAV2/8 transduction of the VMH, as these serotypes are commonly used to transfect the brain.

Working with AAVs has been assigned to Biosafety Level 2 in most countries. For optimal conductance of behavioural experiments, transfer of genetically modified animals to a Biosafety Level 1 laboratory might be required. To indicate when animals injected with AAV can be safely handled at Biosafety Level 1, blood clearance rates were assessed after systemic injection.

## Materials and Methods

### Ethics statement

All experimental procedures were approved by the Committee for Animal Experimentation of the University of Utrecht (Utrecht, the Netherlands).

Human Embryonic Kidney (HEK) 293T cells used in this study were purchased from ATCC.

### Cell lines

HEK293T cells were cultured in growth medium (Dulbecco's modified Eagle medium, DMEM) (Invitrogen, USA) supplemented with 10% fetal calf serum (FCS) (Lonza, Switzerland), 2 mM glutamine (PAA, Germany), 100 units/ml penicillin (PAA, Germany), 100 units/ml streptomycin (PAA, Germany) and non-essential amino acids (PAA, Germany) at 37°C with 5% CO_2_.

### Virus production and purification

Virus was generated and purified as previously described [15]. HEK293T cells were co-transfected with pAAV-LepR (a kind gift from R.J. DiLeone [16]) and pDP1, pDP2, pDP5 or pAR-8+pXX6 (Plasmid factory, Germany) in fifteen 15×15 cm dishes using PEI. Sixty hours after transfection, cells were collected, pelleted and resuspended in ice-cold buffer (150 mM NaCl, 50 mM Tris, pH 8.4). Cells were lysed by three freeze-thaw cycles and incubated for 30 minutes at 37°C with 50 U/ml benzonase (Sigma, The Netherlands). The lysate was loaded onto a 15%, 25%, 40%, and 60% iodixanol gradient. After centrifugation at 70.000 rpm for 60 minutes at 18°C, the 40% layer was extracted and used for ion-exchange chromatography. AAV positive fractions were determined by quantitative PCR (qPCR) on GFP (forward primer 5'- CACATGAAGCAGCACGACTT; reverse primer 5'- GAAGTTCACCTTGATGCCGT) and concentrated using an Amicon Ultra 15 ml filter (Millipore). Titer was determined by qPCR on GFP.

### Animal studies

Male Wistar rats (Charles River, Germany) were housed in filter top cages in a temperature- and humidity-controlled room (temperature 21±2°C and humidity 55±5%) with a 12 h light/dark cycle. AAV2/1, AAV2/2, AAV2/5 or AAV2/8 was administered either stereotactically in the VMH (see: surgical procedure) or systemically via the tail vein. Blood samples from animals injected in the VMH were collected via a tail cut one hour after injection. Blood samples from systemically injected animals were collected 1 m, 10 m, 20 m, 40 m, 1 h, 4 h and 24 h after injection via a tail cut in heparin-coated capillary tubes.

### Surgical procedures

Rats were anesthetized using fentanyl/fluanisone and midazolam and mounted onto a stereotaxic apparatus. Virus was administered by placing a syringe needle into the VMH (coordinates from Bregma: −2.1 AP, +1.5 ML, −9.9 DV, at a 5° angle). A total of 1,5 µl of virus (7×10^11^ genomic copies (gc)/ml AAV2/1, AAV2/5 or AAV2/8; 1.5×10^11^ gc/ml AAV2/2) was injected at a rate of 0.2 µl/minute.

### Viral DNA isolation from blood and real-time PCR

Viral DNA was isolated from blood using the High Pure Viral Nucleic Acid Kit according to the manufacturer's instructions (Roche, Germany). Briefly, 200 µl of binding buffer supplemented with carrier RNA and 50 µl of Proteinase K solution were added to 200 µl of plasma. After mixing, the samples were incubated for 10 minutes at 72°C. 100 µl of binding buffer was added and samples were mixed. Next, samples were transferred to a High Pure Filter Tube and centrifuged at 8000×g for 1 minute. 500 µl Inhibitor Removal Buffer was added to the filter tube followed by centrifugation. The filter was washed twice with 450 µl Wash Buffer followed by centrifugation. To elute viral nucleic acids, 50 µl Elution Buffer was added to the filter tube and samples were centrifuged. PCR was performed on each sample for the GFP gene with the Roche Lightcycler according to the manufacturer's instructions. Viral DNA was assayed for copy number of the GFP gene using the SYBR-Green I (Roche, Germany). AAV plasmid at 1×10^4^ genomic copies (g.c.), 1×10^6^ g.c. and 1×10^8^ g.c. was used as copy number controls. AAV plasmids were dissolved in blood and purified using the High Pure Viral Nucleic Acid Kit (Roche, Germany) as previously described. The lower limit of quantification was 100 gc/µl.

### In situ hybridization

For the in situ hybridization (ISH), cryostat sections of 20 µm thickness from fresh frozen brains were mounted onto slides. Brains were sliced from Interaural: 7.28 mm, Bregma: −1.72 mm to Interaural: 5.64 mm, Bregma: −3.36 mm and the VMH was localized using the atlas ‘The Rat Brain’ (Paxinos and Watson, 6^th^ edition). Sections were fixed in 4% paraformaldehyde (PFA) for 20 minutes, washed in phosphate buffered saline (PBS), acetylated for 10 minutes and washed again. Sections were pre hybridized in hybridization solution (50% formamide, 5× SSC, 5× Denhardts, 250 µg/ml tRNA Baker's yeast, 500 µg/ml sonicated salmon sperm DNA) for 2 hours at room temperature. The hybridization solution containing 400 ng/ml 720 bp long digoxigenin (DIG)-labeled enhanced green fluorescent protein (eGFP) riboprobe (antisense to NCBI gene DQ768212) was then applied to the slides followed by overnight incubation at 68°C. After a quick wash in 68°C pre warmed 2×SSC, slides were transferred to 68°C pre warmed 0.2×SSC for 2 hours. DIG was detected with an alkaline phosphatase labeled antibody (1∶5000, Roche, Germany) after overnight incubation at room temperature using NBT/BCIP as a substrate. Sections were dehydrated in ethanol, cleared in xylene and embedded in Entellan.

ImageJ Software was used to quantify the spread of GFP mRNA expression in the VMH.

### Statistical analyses

All data were presented as means ± SEM. The significance of differences in the comparison of transduction area, blood clearance rate and half-life was evaluated by a Kruskal-Wallis analysis, followed by Mann-Whitney U-test using GraphPad Prism 5 software. A P-value of <0.05 was considered to be significant.

## Results

### Transduction efficiency and biodistribution of AAV2/1, AAV2/5 and AAV2/8

**Figure 1:**
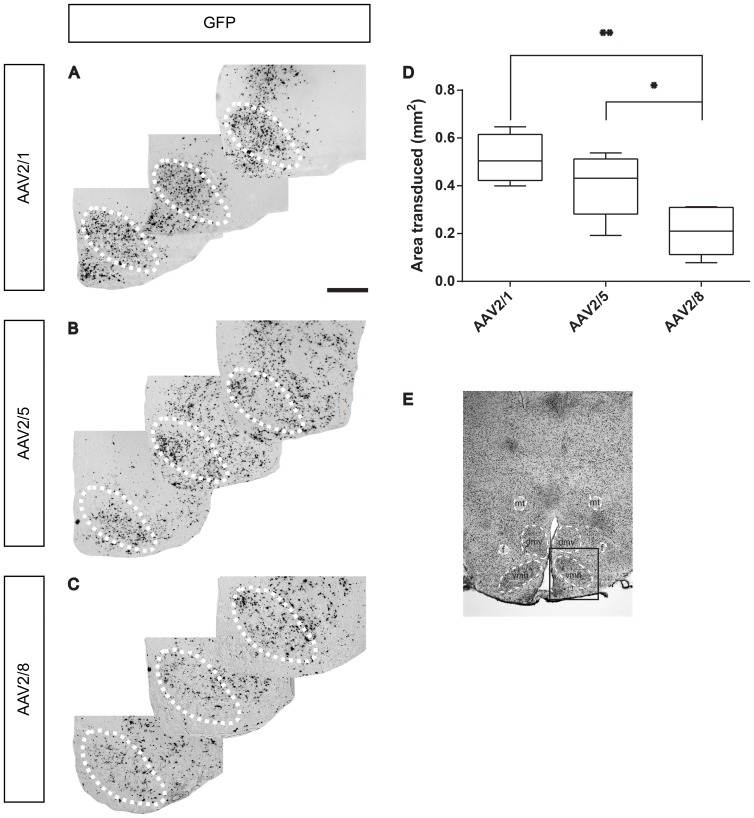
Comparison of transduction efficiency of serotype AAV2/1, AAV2/5 and AAV2/8 in the VMH. Rats (n = 6) were injected with 1×10^9^ genomic copies of AAV2/1, AAV2/5 or AAV2/8 in the VMH. The transduced area was identified using ISH on GFP. AAV2/1 and AAV2/5 were equally efficient in transducing the VMH (A, B, D) and performed significantly better than AAV2/8 (C, D) (P<0.01 and P<0.05, respectively). The VMH area is indicated by a dotted line. Figure E depicts the hypothalamic area. The square indicates the area that is enlarged in Figures A, B and C. mt  =  mammillary tract; f  =  fornix; dmh  =  dorsomedial hypothalamus; vmh  =  ventromedial hypothalamus. Scale bar: 500 µm.

A vector containing AAV2 terminal repeats flanking a shRNA targeting the leptin receptor and an enhanced green fluorescent protein (eGFP) expression cassette was packaged with an AAV1, AAV5 or AAV8 capsid. To determine transduction efficiency, rats (n = 6) received an injection of 1×10^9^ g.c. AAV2/1, AAV2/5 or AAV2/8 in the VMH and their brains were analysed six weeks after injection. AAV2 transduction was not studied for its transduction efficiency as other serotypes have been shown to more efficiently transduce distinct areas of the CNS [3], [4], [8]. A GFP in situ hybridization (ISH) to detect GFP mRNA was performed to precisely identify the transduced area and to analyse transduction efficiency. Both AAV2/1 and AAV2/5 efficiently transduced the VMH (Figure 1a, b and e) and distributed over a significantly larger area (0,51±0,05 mm^2^ and 0,40±0,05 mm^2^, respectively) than AAV2/8 (0,21±0,04 mm^2^) within the VMH (Figure 1c, d and e) (P<0.01 and P<0.05, respectively). No difference in transduction efficiency was observed between AAV2/1 and AAV2/5.

### AAV biodistribution after administration to the brain

To examine AAV biodistribution after virus administration to the brain, serotypes commonly used to transfect the brain were injected into the VMH. Rats (n = 6) received an injection of 3×10^8^ g.c. AAV2/2 or 1×10^9^ g.c. AAV2/1, AAV2/5 or AAV2/8 in the VMH. Blood samples were collected from the tail one hour after virus administration. No genomic copies were detectable by quantitative PCR in blood samples collected one hour after virus administration.

### AAV clearance from blood

**Figure 2:**
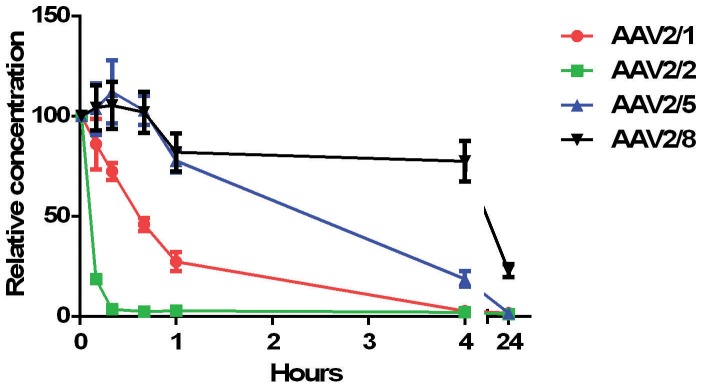
Blood clearance kinetics of AAV2/1, AAV2/2, AAV2/5 and AAV2/8 after systemic administration. Rats (n = 6) received a systemic injection of 1×10^9^ AAV2/1, AAV2/2, AAV2/5 or AAV2/8 and blood samples were collected 10 m, 20 m, 40 m, 1 h, 4 h and 24 h after injection. AAV2/2 showed a significantly faster clearance rate than AAV2/1, AAV2/5 and AAV2/8. Four hours after injection, less than 3% of the starting material of AAV2/1 and AAV2/2 could be traced. AAV2/8 showed a delayed clearance rate. One day after injection still more than 20% of the starting material was present in the circulation.

To quantify the half-life of AAV, blood clearance rates of AAV serotypes commonly used to transfect the brain (AAV1, AAV2, AAV5 and AAV8) were determined. Blood samples were collected 1 m, 10 m, 20 m, 40 m, 1 h, 4 h and 24 h after systemic injection of 1×10^9^ genomic copies into adult rats (n = 6). A very rapid blood clearance rate was found for AAV2/2 compared to the other serotypes (Figure 2). Ten minutes after injection, AAV2/2 exhibited a higher decrease in blood concentration than AAV2/1, AAV2/5 and AAV2/8 (p<0.01). Since there was some variability between rats in plasma AAV concentration one minute after systemic injection, we calculated the relative changes in concentrations. Concentrations below 3% were found for AAV2/1 and AAV2/2 four hours after injection. In contrast, AAV2/8 particles showed a relatively slow blood clearance rate, with a concentration above 10% one day after injection, which is significantly higher than the blood concentration of AAV2/5 (p<0.01).

Half-life was calculated for each serotype (Table 1). A half-life of 4,2 minutes was found for AAV2/2, which was significantly lower than the half-lives of AAV2/1, AAV2/5 and AAV2/8 (P<0.01). AAV2/8 showed a significantly prolonged half-life compared to AAV2/5 (p<0.01).

**Table 1:** Half-life of AAV2/1, AAV2/2, AAV2/5 and AAV2/8.

Serotype	Half life (hours)
1	0.55 ± 0.08
2	0.07 ± 0.00
5	1.67 ± 0.21
8	11.40 ± 1.09

Half-life in blood was calculated after systemic AAV administration in rats (n = 6).

## Discussion

The use of AAV as a tool to manipulate gene expression in the central nervous system has shown much promise. The aim of the present study was to contribute to the efficient and safe use of AAV serotypes commonly used to transduce the mammalian brain.

A comparison of different AAV serotypes in the brain area of interest contributes to the optimization of gene or shRNA transfer. We have already demonstrated that AAV2/1 results in higher levels of gene delivery in the hypothalamic area compared to AAV2/2 and AAV2/8 [3]. This study confirms a more efficient transduction of AAV2/1 compared to AAV2/8 in the VMH. As AAV2/5 is shown to effectively transduce other brain structures [4], [8], [9], this serotype was examined for its transduction efficiency in the VMH. AAV2/5 was found to be equally effective in transducing the VMH as AAV2/1, which corresponds to Burger et al., who compared these serotypes in rat striatum, hippocampus and substantia nigra [4].

The observed divergence in cellular uptake of the AAV serotypes might be explained by their mode of entrance (receptor type) into cells. Cell surface receptors mediating the cellular entry of AAV2 are best known. AAV2 attachment is mediated by heparan sulfate proteoglycans, while co-receptors, including aVb integrin, fibroblast or hepatocyte growth factor receptors and a laminin receptor, enhance internalization [17]–[21]. The laminin receptor is also known to interact with AAV8 [21]. AAV1 and AAV5 lack heparan binding amino acids and subsequently do not use surface heparan sulfate proteoglycans as a receptor for infection [22], [23]. Both utilize sialic acid-containing glycoproteins for efficient binding and transduction [24]–[27]. For AAV5, the platelet-derived growth factor receptor has been shown to serve as a co-receptor [28]. Besides binding and entry, other steps in the AAV infection pathway influence the transduction process, including intracellular viral trafficking, nuclear transport, uncoating and second strand DNA synthesis [29]–[31].

When applying AAV-mediated gene transfer to study the functions of a gene in a certain brain area, it is important to take serotype specific biodistribution into account. If the AAV, which is administered to the brain, is able to cross the blood-brain barrier, its content might exert unwanted side effects in the periphery. It has been reported that AAV2/2 and AAV2/5 are not able to cross the blood-brain barrier both *in vitro* [32] and *in vivo* in mice and rats [14], [33]. However, in nonhuman primates, that received AAV2/2 or 2/5, vector DNA could be traced in the spleen and in the liver and spleen, respectively [34], [35]. No peripheral tissue was transduced in animals receiving lower doses and no genomic copies were detected in the blood [34]. In another study using nonhuman primates, viral DNA was detected in the serum as soon as one hour after intracerebral administration of AAV2/1, 2/2 and 2/5 [36]. Systemically injected AAV2/1 and AAV2/8 have been shown to efficiently cross the blood-brain barrier and subsequently transduce neurons in hypothalamus, cerebellum and spinal cord in the neonatal neonatal mouse brain [14], [37]. This study shows that one hour after administration of AAV2/1, AAV2/2, AAV2/5 and AAV2/8 in the rat VMH no viral particles could be traced in the blood. This indicates that AAVs are not crossing the blood-brain barrier after injection in the brain or that the number is too low to detect by qPCR on blood samples. If the latter is the case, then probably the number of genomic copies crossing is too low to induce an effect. All serotypes have been shown to be able to cross the blood-brain barrier. Interspecies differences in blood-brain barrier composition, titer, age, time after injection and different sites of injection might explain these contradicting outcomes. We cannot rule out that injection with higher titers will result in (detectable) viral particles in the blood.

Blood clearance kinetics of AAV2/1, AAV2/2, AAV2/5 and AAV2/8 were assessed after systemic injection. AAV2/1 and AAV2/2 showed a rapid clearance with less than 3% of the starting material left after 4 hours. This is in accordance to results obtained in mice [38], [39]. However, in contrast to previous results [39], AAV2/8 showed a significantly delayed clearance compared to the other serotypes. The observed differences in AAV serotype clearance might be explained by the number of genomic copies lost to the phagocytic cells in the liver, Kupffer cells, and serotype-specific transcytosis of AAV across the endothelial cells [32], [40].

This study aimed to optimize the safe and efficient use of AAV-mediated gene transfer to the VMH of the rat brain. Efficient transduction of the VMH was achieved using AAV2/1 and AAV2/5. No genomic copies could be found in blood one hour after AAV administration to the brain and both serotypes exhibit a relatively fast clearance from blood. This minimizes the risk for transduction of peripheral organs, which could potentially influence phenotypic results.
